# Effect of blood contamination of cerebrospinal fluid on amino acids, biogenic amines, pterins and vitamins

**DOI:** 10.1186/s12987-019-0154-5

**Published:** 2019-11-14

**Authors:** Marta Batllori, Mercedes Casado, Cristina Sierra, Maria del Carmen Salgado, Laura Marti-Sanchez, Joan Maynou, Guerau Fernandez, Angels Garcia-Cazorla, Aida Ormazabal, Marta Molero-Luis, Rafael Artuch

**Affiliations:** 1 Clinical Biochemistry Department, Hospital Sant Joan de Déu, Barcelona, Spain; 2 Molecular Genetics, Hospital Sant Joan de Déu, Barcelona, Spain; 3 Pediatric Neurology Department, Institut de Recerca Sant Joan de Déu, Barcelona, Spain; 4 CIBERER-Instituto de Salud Carlos III, Barcelona, Spain

**Keywords:** Cerebrospinal fluid, Amino acids, Biogenic amines, Pterins, Vitamins, Blood contamination

## Abstract

**Background:**

Cerebrospinal fluid (CSF) metabolomic investigations are a powerful tool for studying neurometabolic diseases. We aimed to assess the effect of CSF contamination with blood on the concentrations of selected biomarkers.

**Methods:**

CSF samples were spiked in duplicate with increasing volumes of whole blood under two conditions: (A) pooled CSF spiked with fresh whole blood and frozen to cause red blood cell (RBC) lysis; (B) pooled CSF spiked with fresh blood and centrifuged (the supernatant with no RBCs was frozen until the moment of analysis). CSF concentrations of amino acids, biogenic amines, pterins, and vitamins were analysed by HPLC coupled with tandem mass spectrometry, electrochemical and fluorescence detection.

**Results:**

Aspartate, glutamate, taurine, ornithine, glycine, citrulline, pyridoxal 5´-phosphate, 5-methyltetrahydrofolate, and thiamine showed higher values when RBCs were lysed when compared with those of CSF with no RBC, while arginine, 5-hydroxyindoleacetic and homovanillic acids showed lower values. When RBCs were removed from CSF, only some amino acids, thiamine and pyridoxal 5´-phosphate showed moderately higher values when compared with the non-spiked CSF sample.

**Conclusions:**

CSF-targeted metabolomic analysis is feasible even when substantial RBC contamination of CSF has occurred since CSF centrifugation to remove RBC prior to freezing eliminated most of the interferences observed.

## Background

Cerebrospinal fluid (CSF) is a biological fluid that is mainly produced by the choroid plexus, which constitutes the interface between blood vessels and CSF [1, 2]. The composition of CSF is also controlled by the blood–brain barrier, which separates blood from the brain parenchyma [3]. Both structures deliver substrates for brain cell metabolism and remove the corresponding waste [1, 4, 5]. In general, the blood–brain barrier greatly restricts the influx of most molecules, including amino acids and other compounds [6]. Amino acids, with few exceptions (e.g., glutamine), show lower values in CSF when compared with those of plasma [7, 8]. For other metabolites, biosynthetic pathways are compartmentalized in the brain, and similar concentrations may be observed in CSF and blood since no transport from blood to CSF is expected; this is the case for biogenic amines and pterins [9, 10]. In contrast, some vitamins have to be transported into the brain through central nervous system barriers by specific transporters, and differences between vitamin concentrations in CSF and blood samples are noticeable [11, 12]. While folate is one of the few molecules more concentrated in CSF when compared to plasma, other vitamins such as thiamine and pyridoxine display lower values in CSF when compared to those of blood [12, 13].

CSF metabolomic investigations have been demonstrated to be a powerful tool for studying specific neurometabolic pathways and related diseases and for exploring metabolic transport from the blood into the brain [14]. Several neurogenetic conditions are caused by specific disturbances in these processes (Table 1). In recent decades, targeted metabolomic approaches have been used for the study of these neurogenetic conditions [14]. Owing to the important differences in the metabolite concentrations between blood and CSF, contamination of CSF with blood may cause dramatic effects in the measured concentrations of most of the above-mentioned metabolites [15–18]. CSF is collected by lumbar puncture, which is an invasive method. Since blood/plasma contamination can be frequently observed by different causes (traumatic lumbar punctures, impaired blood–brain barrier permeability or intraventricular bleeding) [19–21], a misinterpretation of metabolic profiles is a problem that should be minimized to avoid repeated lumbar puncture procedures and diagnostic errors.

**Table 1:** CSF biomarkers and their semiological value for different diseases (left columns)

Biomarkers	Diseases and expected value: high or low (↑/↓)	A). Blood/CSF ratio mean value of CSF pools 1 and 2 (standard error of the mean)	B). Plasma/CSF ratio mean value of CSF pools 1 and 2 (standard error of the mean)
Albumin		28 (3.9)	29 (3.2)
Amino acids			
Taurine	–	4.9 (0.2)	2.9 (0.5)
Aspartate	–	15 (8.2)	1.3 (0.07)
Threonine	–	1.9 (0.1)	1.7 (0.2)
Serine	Serine deficiency (↓)	1.6 (0.1)	1.3 (0.1)
Glutamate	–	7.3 (1.7)	1.6 (0.1)
Glutamine	Hyperamoniemias (↑)	1.05 (0.03)	1.1 (0.1)
Glycine	Hyperglycinemias (↑)	3.2 (0.06)	2.2 (0.1)
Alanine	Mitochondrial Dis. (↑)	2.5 (0.3)	2.5 (0.4)
Citrulline	–	2.6 (0.2)	2.1 (0.3)
Valine	BCAA defects (↑/↓)	2.5 (0.3)	2.6 (0.5)
Methionine	–	2.4 (0.3)	2.5 (0.4)
Isoleucine	BCCA defects (↑/↓)	2.3 (0.2)	2.3 (0.3)
Leucine	BCCA defects (↑/↓)	2.3 (0.3)	2.2 (0.4)
Tyrosine	–	2.05 (0.2)	1.8 (0.2)
Phenylalanine	–	1.4 (0.1)	1.6 (0.2)
Ornithine	–	4.6 (0.6)	1.9 (0.02)
Lysine	–	2.2 (0.3)	2.2 (0.3)
Hystidine	–	1.9 (0.01)	1.8 (0.1)
Arginine	–	0.55 (0.1)	1.5 (0.1)
5-HIAA	Serotonin related (↓)	0.6 (0.04)	0.93 (0.04)
HVA	Dopamine (↑/↓)	0.78 (0.01)	0.9 (0.01)
Biopterin	Pterin defects (↑/↓)	1.0 (0.1)	0.9 (0.1)
Neopterin	Pterin defects (↑/↓) Immune events (↑)	1.1 (0.1)	1.1 (0.1)
Thiamine	Transport defects (↓)	3.0 (0.05)	2.2 (0.05)
5-MTHF	Transport/metabolism defects (↓)	1.75 (0.1)	1.0 (0.1)
PLP	Transport/metabolism defects (↓)	3.7 (0.3)	2.5 (0.3)

Blood/CSF ratios for the two experimental conditions: A) Red blood cells (RBC) lysed in CSF and B) RBC removed from CSF. CSF samples were spiked with 20% of whole blood. A total of 20 CSF aliquots coming from 50 CSF samples were analysed (see details in Additional file 2: Figure S1)

When CSF blood contamination occurs, the most critical metabolites for data interpretation can be glycine (the ratio blood/CSF glycine values is very high) and vitamins such as pyridoxine, thiamine and folate: In genetic diseases leading to brain pyridoxine, folate, and thiamine deficiencies, the blood concentrations of these vitamins can be normal, while CSF values may be near undetectable. Thus, blood contamination could mask the CSF vitamin deficiency. The monoamines HVA and 5-HIAA are sensitive to haemoglobin oxidation

*BCAA* Branched chain amino acids

With this background, we aimed to assess the effect of CSF contamination with blood on the concentrations of selected molecules which are biomarkers for the study of different neurometabolic conditions.

## Methods

### Samples

CSF samples were collected from patients where lumbar puncture was done to rule out meningoencephalitis, and stored at − 80 °C, following a previously reported protocol [22]. The remnants of 50 CSF anonymized samples with no red blood cell (RBC) contamination (assessed by light microscopy as less than 5 RBC per field) were thawed, pooled (25 samples for pool 1 and the other 25 for pool 2), reaching a final volume of 10 mL for each pool. The pooled samples were divided into 1 mL aliquots, which were spiked with different volumes of whole blood (at that moment, a fresh blood sample was withdrawn from a healthy volunteer). Non-spiked CSF samples and four spiking conditions were prepared in duplicate in the 2 CSF pools. The CSF pools were spiked with increasing volumes of whole blood: 2.5%, 5%, 10%, and 20%, in 2 different conditions: (A) CSF samples spiked with fresh whole blood and then frozen at − 80 °C to cause RBC lysis. (B). CSF samples spiked with fresh blood, then centrifuged at 1500×*g* 10 min at 4 °C, with the clear supernatant frozen at − 80 °C. Details of the protocol are stated in Additional file 1: Table S1 and Additional file 2: Figure S1. The total sample preparation time spent was 45 min (all samples were frozen at the same time). With these conditions we could assess the effect of whole blood interference (RBC can increase metabolite concentrations in CSF and can cause oxidative/catabolic effects on some of the metabolites studied (condition A) when compared with plasma contamination (condition B), where an increase in metabolites which are more concentrated in plasma than CSF is expected).

Initially, to identify a cut-off value at which blood contamination can cause substantial interference in the measurement of the above-mentioned metabolites, CSF was spiked with whole blood volume range from 1 to 0.02%. No relevant effects were detected in most metabolite concentrations studied under these conditions (data not shown).

### Methods

The concentration of albumin in CSF, used as a surrogate biomarker of CSF RBC contamination or impaired blood–brain and blood-CSF barriers, was analysed using an Abbot automated analyser (Architect c8000) by spectrophotometric procedures. Other biomarkers of blood contamination such as haemoglobin concentration were analysed by an automated procedure (Advia 2120, Siemens Diagnostics). CSF amino acids were analysed by UHPLC coupled to tandem mass spectrometry detection in a Xevo QT Waters system, as previously reported [23]. Biogenic amines (5-hydroxyindoleacetic (5-HIAA) and homovanillic (HVA) acids) and pterins (biopterin and neopterin) were analysed as biomarkers of serotonin and dopamine deficiencies (and in the case of neopterin, also as a biomarker of neuroinflammatory conditions) by HPLC with electrochemical and fluorescence detection as previously reported [22]. The vitamins thiamine, thiamine-diphosphate (TDP), 5-methyltetrahydrofolate (5-MTHF) and pyridoxal 5´-phosphate (PLP) were analysed by HPLC with fluorescence detection as reported [14, 22, 24]. Typical chromatograms of these procedures are presented in Additional file 3: Figure S2.

### Data analysis

The precision of the different techniques was initially calculated using the coefficient of variation (CV = standard deviation/average × 100%) from 20 replicates and was below 10% for all of the metabolites studied, as previously reported [22–24]. Thus, we considered that the effect of blood contamination on CSF samples was not significant when it was lower than 10% when compared with the value obtained in the non-spiked CSF samples. CSF parameters studied here are accredited by the ENAC (ISO 15,189 norm) and certified by AENOR agencies (ISO 9001 norm). CSF amino acids, pterins, and biogenic amines are subjected to external quality control schemes from ERNDIM (data of the results available on request).

### Ethical issues

CSF anonymized samples from remnants were collected in our Hospital following our diagnostic protocols, and the study was conducted only once such investigations were concluded. In every case, informed consent was obtained from each patient before performing the lumbar puncture and CSF collection. The Ethical committee of Sant Joan de Déu Hospital approved the study. All samples from the patients were obtained following the 2013 revised Helsinki Declaration of 1964.

## Results

**Fig. 1:**
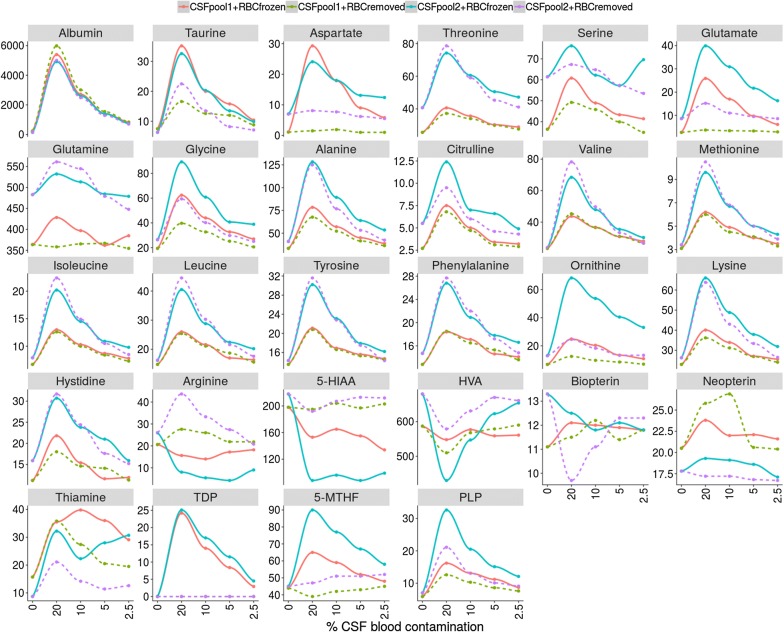
Graphical representation of the concentrations of the different biomarkers analysed in non-spiked CSF samples, and in CSF samples spiked with 20%, 10%, 5% and 2.5% of whole blood, respectively. Albumin is expressed in g/L, amino acids in µmol/L and biogenic amines, pterins and vitamins in nmol/L

**Fig. 2:**
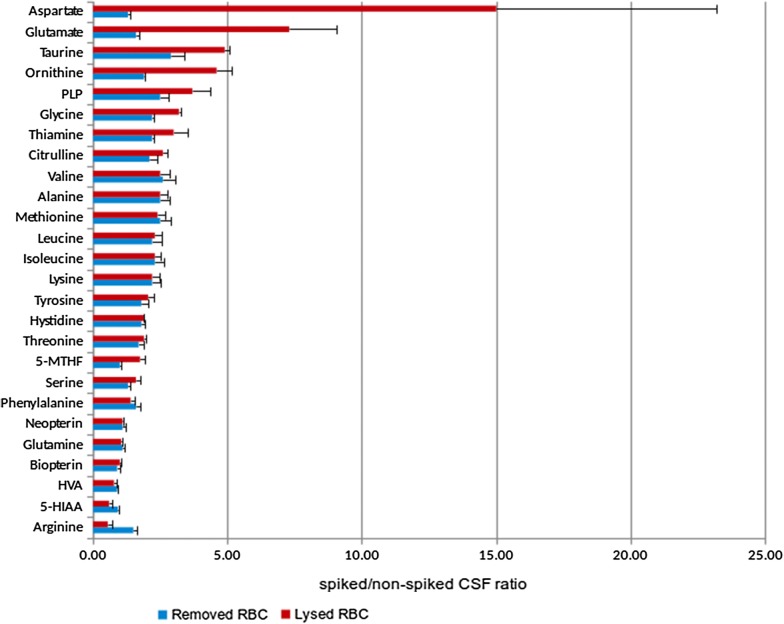
Horizontal bar representation of the differences between spiked/non-spiked CSF ratio of all metabolites in the two experimental conditions. **a** Red bar: red blood cells (RBC) lysed before CSF freezing. **b** Blue bar: RBC removed from CSF by centrifugation before freezing. CSF samples were spiked with 20% of whole blood

The absolute values of the different metabolites studied are depicted in Fig. 1. In Table 1, a list of the metabolites analysed is presented together with the related neurological diseases, the interpretation of a change in the metabolite concentrations and the mean differences in the expected concentration between blood and CSF when CSF was contaminated with 20% of blood under the 2 different experimental conditions designed: condition A: lysed RBC; condition B: removed RBC. Figure 2 is a horizontal bar representation of the differences stated in Table 1. Aspartate, glutamate, taurine, ornithine, glycine, and citrulline had higher values when RBC were lysed when compared with RBC removed from CSF before freezing. PLP, 5-MTHF, and thiamine also showed this tendency. Arginine, 5-HIAA, and HVA had lower values when RBC were lysed in the CSF samples, while the rest of metabolites studied were consistent between the two different conditions.

In Table 2 and Fig. 1 data from the different spiking conditions (represented as the percentage of variation and absolute values when compared with non-spiked CSF samples, respectively) are shown. The 2.5% spiking condition A (lysed RBC) still had percentages of variation moderately higher than 10% for most metabolites. However, in the spiking condition B (RBC removed from CSF), only some amino acids, thiamine and PLP had variations higher than 10% when compared with the non-spiked CSF sample values. As expected, albumin was still highly elevated under all conditions. TDP, an intracellular form of thiamine, was only increased under the spiking condition A, but undetectable when RBC were removed from CSF by centrifugation. While most metabolites displayed changes in concentrations when RBCs were added to CSF, arginine, 5-HIAA and HVA had decreased values. Pterins and glutamine concentrations did not vary under the different spiking conditions.

**Table 2:** Percentage of variation of the different biomarkers measured according to the different volumes of blood spiked into the CSF (expressed as percentage) when compared with the non-spiked CSF samples

Biomarkers (% blood contamination)	A) RBC lysed				B) RBC removed			
	20%	10%	5%	2.5%	20%	10%	5%	2.5%
Albumin	2724	1372	667	323	2877	1379	669	318
Taurine	393	194	112	48	189	91	45	14
Aspartate	1404	842	401	252	42	25	< 10	< 10
Threonine	70	43	21	14	69	38	14	< 10
Serine	45	18	13	< 10	22	15	< 10	< 10
Glutamate	611	394	205	107	57	27	16	< 10
Glutamine	14	< 10	< 10	< 10	< 10	< 10	< 10	< 10
Glycine	229	114	61	43	114	60	21	< 10
Alanine	174	95	46	23	154	72	30	11
Citrulline	151	56	22	< 10	112	42	20	< 10
Valine	138	79	40	23	161	83	36	13
Methionine	141	78	38	20	151	73	39	11
Isoleucine	125	68	34	19	136	69	29	< 10
Leucine	113	62	27	17	123	65	30	< 10
Tyrosine	84	43	20	11	87	41	19	< 10
Phenylalanine	63	38	18	11	66	39	18	< 10
Ornithine	346	256	154	107	86	41	14	< 10
Lysine	113	66	31	16	100	50	22	< 10
Hystidine	93	44	18	< 10	80	42	18	< 10
Arginine	− 47	− 56	− 51	− 39	51	27	< 10	< 10
5-HIAA	− 42	− 37	− 41	− 44	< 10	< 10	< 10	< 10
HVA	− 22	− 11	< 10	< 10	< 10	< 10	< 10	< 10
Biopterin	< 10	< 10	< 10	< 10	< 10	< 10	< 10	< 10
Neopterin	12	< 10	< 10	< 10	< 10	< 10	< 10	< 10
Thiamine	198	154	175	169	135	68	37	27
5-MTHF	74	52	33	19	< 10	< 10	< 10	< 10
PLP	270	157	102	60	157	80	45	29
Hemoglobin (g/dL)	3.02	1.55	0.76	0.37				
TDP (nmol/L)	25	14	11	4.5				

Data are expressed as the mean value of the 2 CSF pools from the 2 conditions: A) spiked CSF frozen containing lysed-RBC cells (left columns), and B) spiked CSF with RBC removed by centrifugation prior freezing (right columns). A percentage of variation below 10% (comparing spiked CSF samples to the non-spiked CSF samples) was considered as the cut-off value for a proper interpretation of the results, considering the coefficient of variation for every biomarker measured when procedures were standardized. Haemoglobin and TDP values are expressed in g/dL and nmol/L, respectively since their values in condition B were not detectable. A total of 20 CSF aliquots coming from 50 CSF samples were analysed (Additional file 2: Figure S1)

Results of the surrogate biomarkers of CSF blood contamination (albumin and haemoglobin) are stated in Additional file 1: Table S1 and Additional file 2: Figure S1.

## Discussion

CSF metabolomic analysis is a good analytical tool for the study of the neurometabolic conditions stated here [14]. Furthermore, in such diseases, the quantification of these metabolites in blood/urine is not reliable, because they usually display normal or even paradoxical results [25]. This is especially true when the metabolic pathways studied are highly active in the brain, or the genetic blood–brain barrier transport diseases related to vitamins and other metabolites.

CSF RBC contamination is frequent and has been recognized as a substantial confounding factor for proper interpretation of CSF analysis data describing concentrations of amino acids and other molecules [26, 27]. However, literature regarding blood contamination effects on biogenic amines, pterins and vitamins is scarce [28]. The main causes of CSF blood contamination are traumatic lumbar punctures or spontaneous intrathecal bleeding, which can occur in several situations, especially in newborns. Moreover, impaired blood–brain barrier permeability can occur under different conditions, such as in asphyxia and epilepsy [19–21]. Thus, having an estimation of when a misinterpretation of the metabolic profile can occur due to RBC/plasma contamination is important, considering that lumbar puncture is an invasive intervention, that it is difficult to perform, and that the final volume collected is sometimes low in paediatric patients.

Albumin, a protein synthesized in the liver, is a good surrogate biomarker for compromised permeability of the blood–brain barrier and also for blood contamination. However, since its concentration in the blood largely exceeds that of the CSF (by approximately 100-fold), its concentration may remain elevated even in the case of low RBC/plasma contamination (in our hands around 1% of blood contamination; data not shown). Haemoglobin measures are an alternative surrogate marker when RBC lysis has occurred, and in our hands, values around 0.35 g/dL of haemoglobin may be a signal for cautious interpretation of the data presented here (Table 2), since it corresponds to a CSF blood contamination approximately from 2.5%, which is the limit where some metabolites may display artefactual results after haemolysis.

With regards to amino acids, several reports have indicated differences between blood and CSF compartments [8], but to our knowledge precise definitions of the limits under which RBC contamination can cause a misinterpretation of the metabolic profiles were not established. While lysed RBCs affected the concentrations of most amino acids at 2.5% of blood contamination, centrifugation of the spiked CSF samples to remove RBC resolved the problem in most cases. In any case, the amino acids that had higher values were aspartate, glutamate, threonine, ornithine, glycine and citrulline. The explanation for this is that some of these amino acids display higher concentration in RBC when compared with plasma (aspartate, glutamate and threonine) [8]. Amongst the other amino acids, glycine has the highest plasma/CSF ratio [29]. Regarding ornithine, arginase activity is high in RBC, and this would explain our observation that arginine values were lower when RBC lysis occurred while ornithine values increased, as it is the product of the reaction catalysed by arginase [8]. Once again, centrifugation of the samples prevented all of these contamination biases. Some of the results in Table 2 were striking, however. No precise correlations with the expected values were observed for some amino acids considering the different percentages of blood contamination. A possible explanation is that some amino acids are present in CSF at very low concentrations physiologically (close to the quantification limits) and for other amino acids, the matrix effect, common in UPLC-MS/MS technology, could contribute to such differences [22].

Biogenic amines and pterins are synthesized peripherally in some tissues but also in the brain, and no substantial transport has been documented between blood and brain (only OAT3 transporters efflux both 5-HIAA and HVA from CSF to blood [2]). Since their concentrations in blood are similar to those of CSF [9, 10], no significant changes were observed after CSF blood contamination. Interestingly, both 5-HIAA and HVA had lower values when RBC lysis was caused. Autoxidation of these molecules by haemoglobin/free radicals is a potential mechanism explaining this observation [30], and thus, one should be cautious when interpreting data when CSF has not been centrifuged prior freezing, since low 5-HIAA and HVA values are surrogate biomarkers of serotonin and dopamine deficiencies and may be an indication of therapeutic intervention [31]. In any case, centrifugation and RBC removal prior to freezing corrected the results when compared with non-spiked CSF.

Vitamins displayed unpredictable results, except for folate. Folate forms (especially 5-MTHF) are highly concentrated in RBC when compared with plasma and this would explain the positive interference observed when RBC lysis occurred, but not after RBC removal. Only thiamine and PLP had increased values when comparing spiked CSF samples at 2.5% with non-spiked samples under both experimental conditions, although it was less remarkable when RBC removal was performed. These effects were minimized when the CSF blood contamination was 1% (data not shown). Regarding thiamine, active conversion of free thiamine, TMP and TDP occur inside cells (RBCs have a high activity of either thiamine phosphokinase, which phosphorylates thiamine to form TDP, or thiamine phosphatases, which convert TDP to TMP and thiamine) [32]. This would explain the plateau results observed when RBC lysis occurred, results that were minimized when RBCs were removed. In any case, thiamine values are higher in blood than in CSF, and thus, the results should be cautiously interpreted when RBC contamination occurs [12]. TDP, a strictly intracellular thiamine vitamer [32], would be a good surrogate biomarker of RBC lysis in CSF samples since undetectable amounts of TDP were observed when RBCs were removed from CSF. With PLP, the observations were similar, and although less significant, even when RBCs were removed from CSF, PLP displayed higher concentrations in the spiked CSF samples. As with thiamine, a complex intracellular metabolic pathway accounts for the synthesis of the different pyridoxine-related vitamers [13]. Moreover, some of these vitamins can be degraded by nucleophiles and oxygen-derived free-radicals, as CSF has low concentrations of other molecules that can react with these compounds [13]. Thus, results should be analysed cautiously concerning to these two vitamins, since either in thiamine or pyridoxine related disorders, which cause severe neurological phenotypes, diagnostic hallmarks are low CSF thiamine and PLP values [12, 13, 33].

## Conclusions

CSF-targeted metabolomic analysis is feasible even when remarkable RBC CSF contamination occurs since CSF centrifugation to remove RBC prior to freezing prevents most of the biases observed. However, data should be cautiously interpreted, especially for some metabolites. CSF albumin, haemoglobin, and TDP can be used as surrogate biomarkers of the potential confounding effect of CSF plasma/RBC contamination.

## Supplementary information

**Additional file 1: Table S1.** Percentage of blood contamination, albumin and haemoglobin levels from CSF spiked with increasing amounts of blood.

**Additional file 2: Figure S1.** CSF blood spiking protocol. A total of 20 CSF aliquots were analysed. In the picture, the colours of the 5 CSF sample are presented. Even in the 2.5% spiking condition, the red colour was intense. The median CSF blood contamination observed in our laboratory typically ranged from 0.01 to 0.035 g/dL of a haemoglobine, which is lower than the 0.35 g/dL observed in the 2.5% blood contamination condition.

**Additional file 3: Figure S2.** Typical chromatograms of the different metabolites analysed in non-spiked CSF samples: (1) Amino acids. (2) Biogenic amines. (3) Pterins. (4) 5-methyltetrahydrofolate. (5) Pyridoxal 5´-phosphate. (6) Thiamine.

## Data Availability

The datasets used and/or analysed during the current study are available from the corresponding author on reasonable request.

## Abbreviations

CSF: cerebrospinal fluid
RBC: red blood cells
UHPLC: ultra-high-performance liquid chromatography
5-HIAA: 5-hydroxyindoleacetic acid
HVA: homovanillic acid
HPLC: high-performance liquid chromatography
TDP: thiamine-diphosphate
5-MTHF: 5-methyltetrahydrofolate
PLP: pyridoxal 5´-phosphate
BCAA: branched-chain amino acids
