# Supplementary material for: Effect of blood contamination of cerebrospinal fluid on amino acids, biogenic amines, pterins and vitamins
Source: Fluids Barriers CNS. 2019 Nov 14;16:34. doi: 10.1186/s12987-019-0154-5 (PMC6857153; doi:10.1186/s12987-019-0154-5)

Additional file 3: Figure S2

1) Amino acid

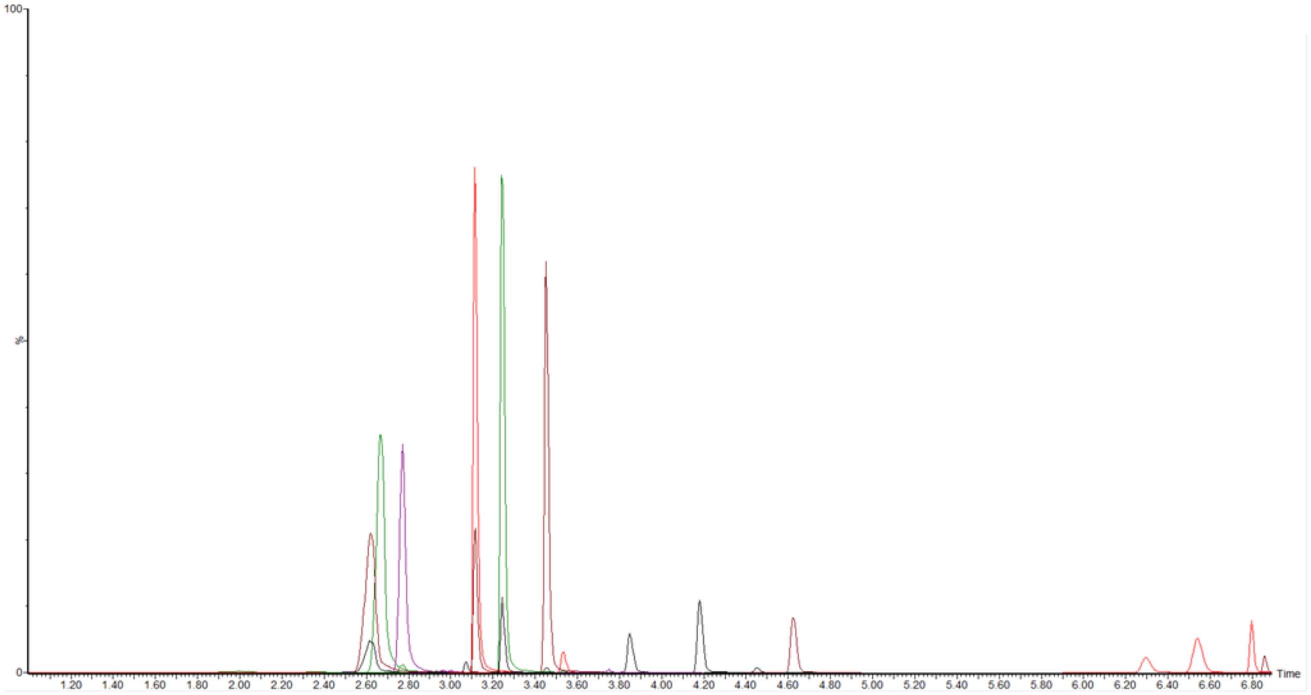

2) Biogenic amines

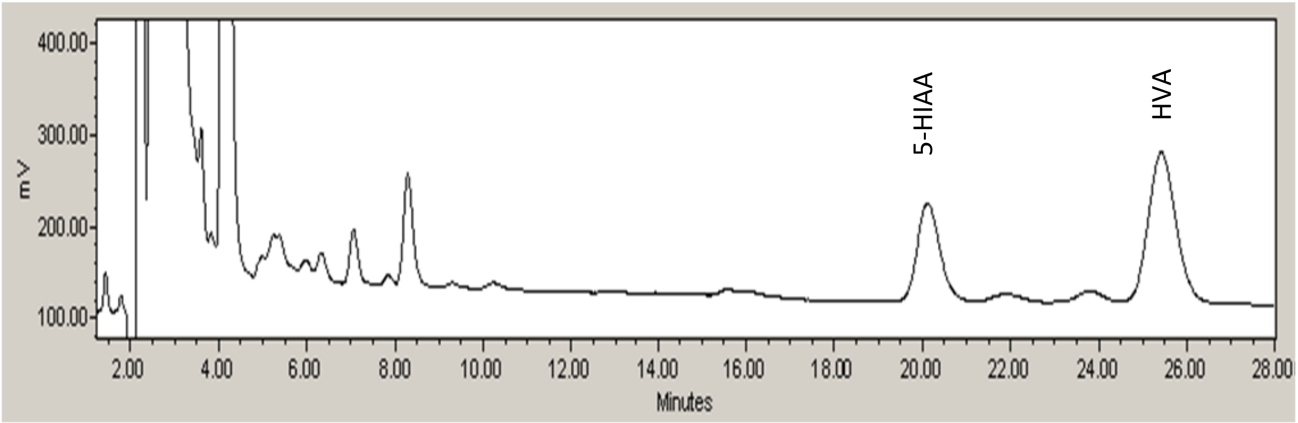

3) Pterins

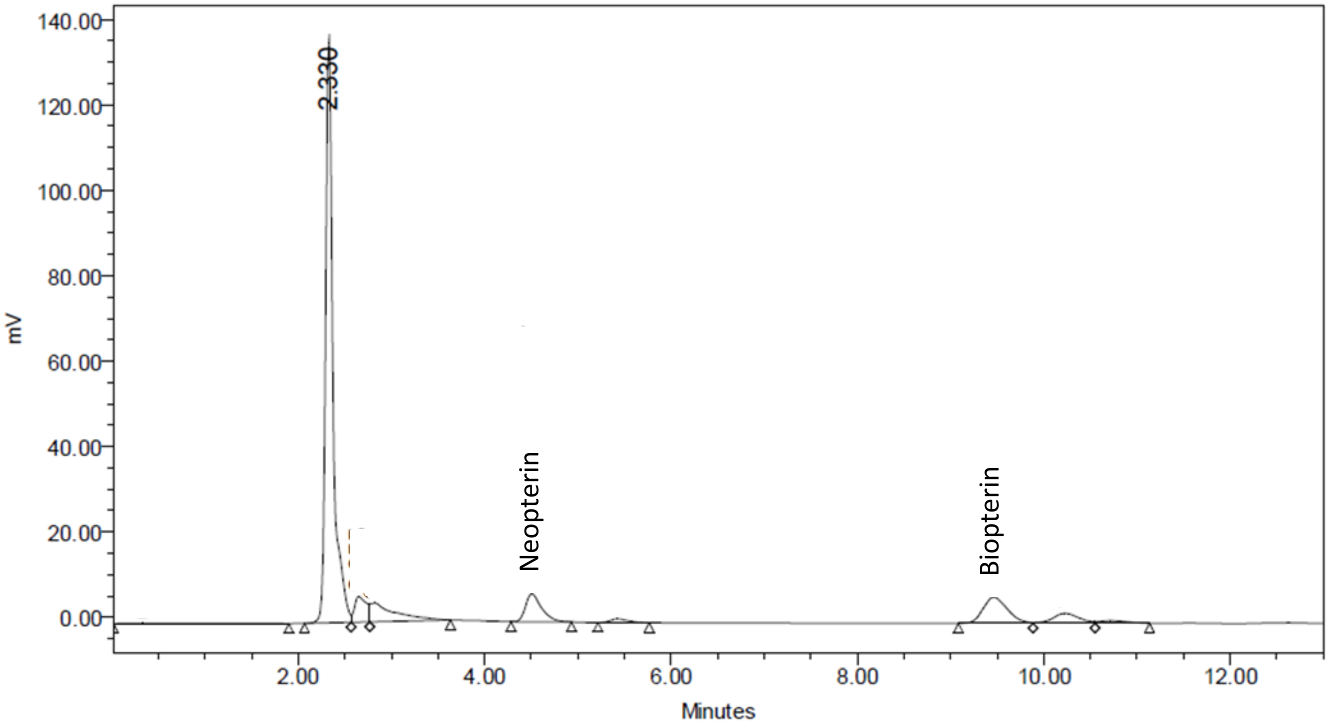

4) 5-methyltetrahydrofolate

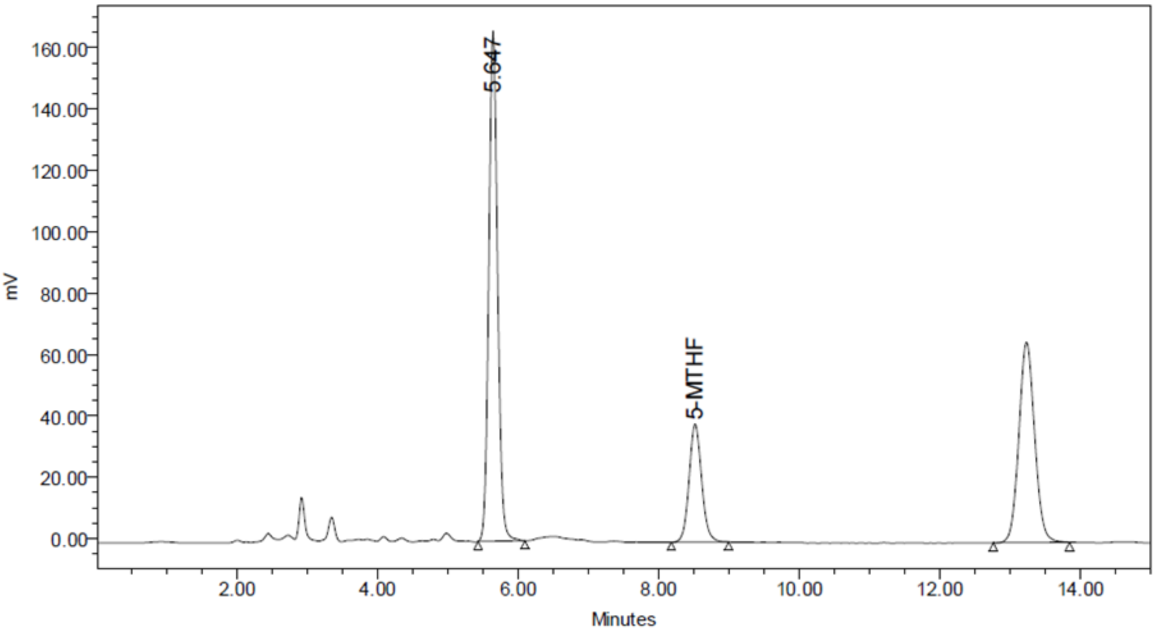

5) Pyridoxal 5'-phosphate

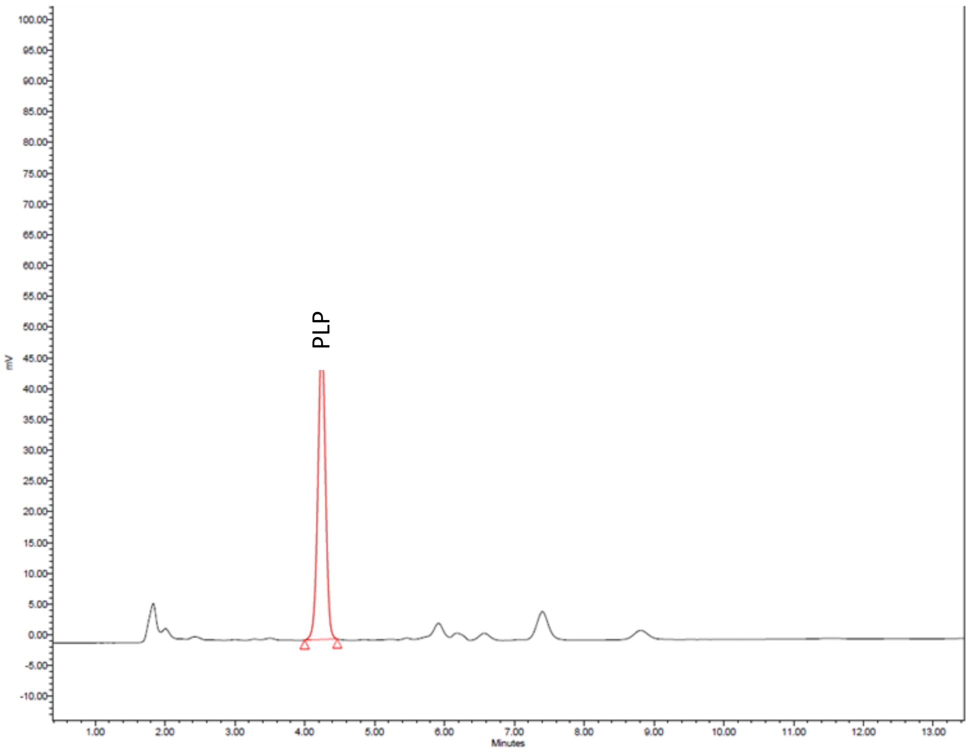

6) Thiamine

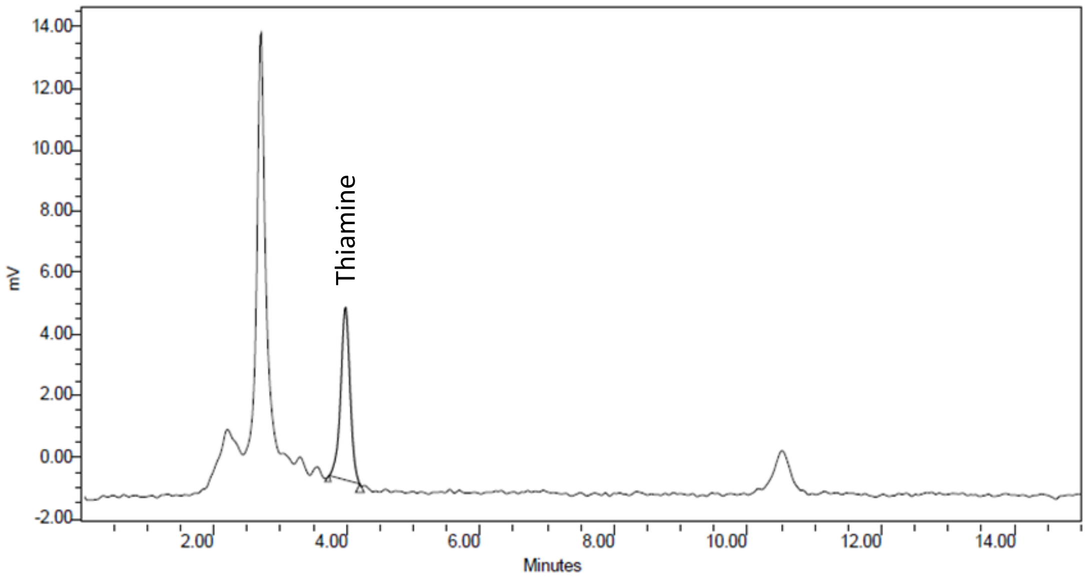

Supplement: Supplementary file 1 — Additional file 1: Table S1. Percentage of blood contamination, albumin and haemoglobin levels from CSF spiked with increasing amounts of blood. [file 12987_2019_154_MOESM3_ESM.pdf]
