# Supplementary material for: Effect of blood contamination of cerebrospinal fluid on amino acids, biogenic amines, pterins and vitamins
Source: Fluids Barriers CNS. 2019 Nov 14;16:34. doi: 10.1186/s12987-019-0154-5 (PMC6857153; doi:10.1186/s12987-019-0154-5)
Supplement: Supplementary file 2 — Additional file 2: Figure S1. CSF blood spiking protocol. A total of 20 CSF aliquots were analysed. In the picture, the colours of the 5 CSF sample are presented. Even in the 2.5% spiking condition, the red colour was intense. The median CSF blood contamination observed in our laboratory typically ranged from 0.01 to 0.035 g/dL of a haemoglobine, which is lower than the 0.35 g/dL observed in the 2.5% blood contamination condition. [file 12987_2019_154_MOESM1_ESM.docx]

| Whole blood (µL) | 200 | 100 | 50 | 25 |
| --- | --- | --- | --- | --- |
| Cerebrospinal fluid (µL) | 800 | 900 | 950 | 975 |
| % of blood contamination | 20% | 10% | 5% | 2.5% |
| Albumin levels (g/L) | 9.2 | 4.6 | 2.3 | 1.15 |
| Haemoglobin levels (g/dL) | 2.9 | 1.45 | 0.72 | 0.35 |

**Additional file 1: Table S1**
