# Supplementary figures and images for: Effect of blood contamination of cerebrospinal fluid on amino acids, biogenic amines, pterins and vitamins
Source: Fluids Barriers CNS. 2019 Nov 14;16:34. doi: 10.1186/s12987-019-0154-5 (PMC6857153; doi:10.1186/s12987-019-0154-5)

Additional file 2: Figure S1

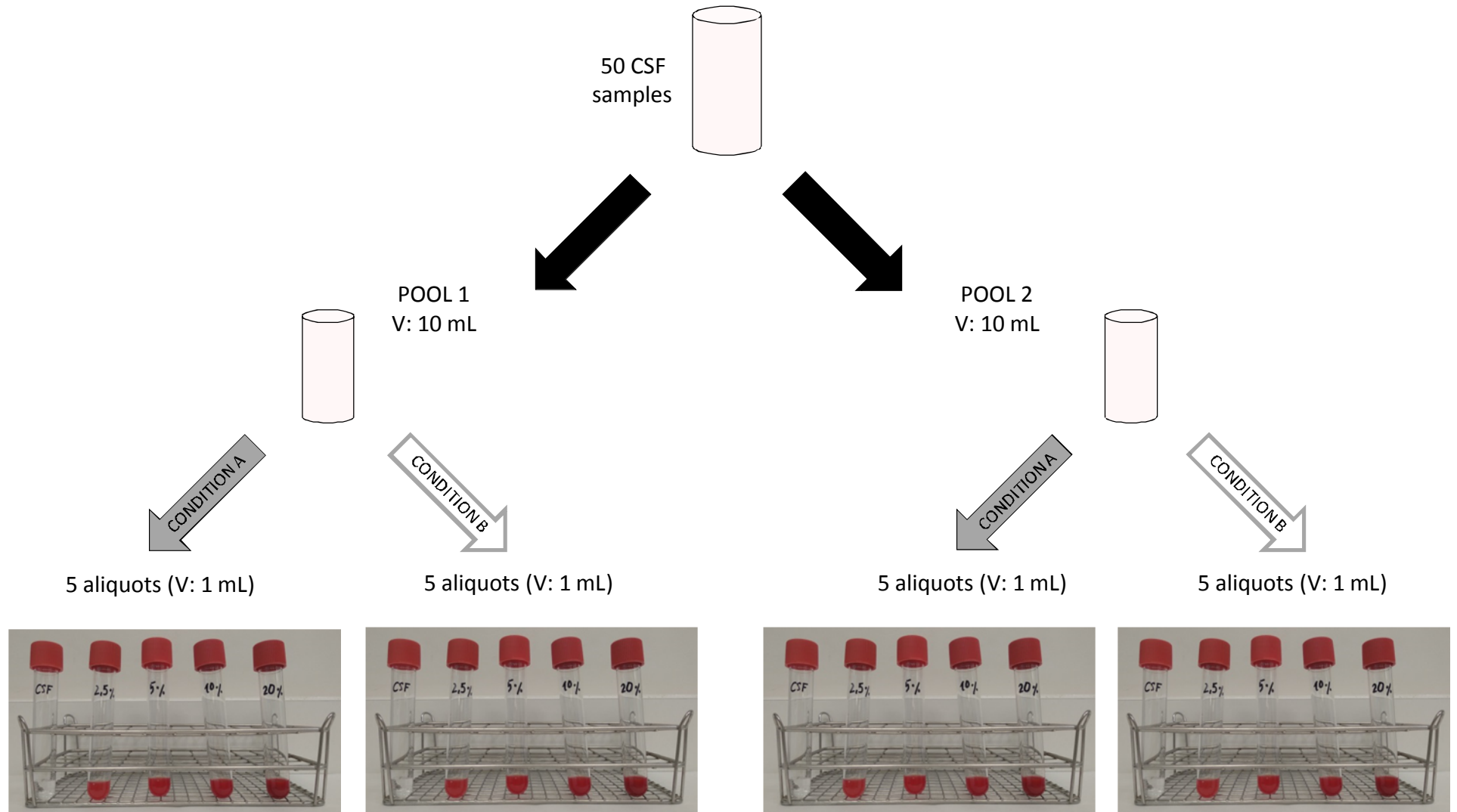

Supplement: Supplementary file 3 — Additional file 3: Figure S2. Typical chromatograms of the different metabolites analysed in non-spiked CSF samples: (1) Amino acids. (2) Biogenic amines. (3) Pterins. (4) 5-methyltetrahydrofolate. (5) Pyridoxal 5´-phosphate. (6) Thiamine. [file 12987_2019_154_MOESM2_ESM.pdf]
